# Book on the Physician Himself and Things That Concern His Reputation and Success

**Published:** 1889-09

**Authors:** 


**Book on the Physician Himself and Things that Concern His Reputation and Success.** By Dr. W. CATHELL, M. D., Baltimore, Md. The ninth edition, revised and enlarged. 8vo, cloth; pp. 298. Philadelphia and London: F. A. Davis, publisher. 1889.

We do not believe the profession needs a book like this, and, therefore, regret its having been published. That it has reached a ninth edition, and thus shown that it is successful from a business standpoint, may excuse but does not justify the author. A physician who thinks and lives properly, does not need this book; one who does not so live and think, will not do so because of it. Much of the advice is very good, but good advice is not more apt to make a physician either good or great than one swallow is to make a summer. The book is really a code of ethics gone mad. Every situation the author can think of, or hear about, is detailed, with the proper line of conduct suggested. We submit that the best possible book written in this way will fail, as the enormous mass of detail will obscure the real end desired. The work appears in a pleasant shape; is finely printed on good paper, and is a credit to the publisher. A good index completes it. F. H. P.

